# Impact of Blood Donation Frequency on the Prevalence of Cardiovascular Risk Factors Among Donors

**DOI:** 10.1155/ghe3/6687660

**Published:** 2026-08-17

**Authors:** Collince Odiwuor Ogolla, Bernard Guyah, Apollo O. Maima

**Affiliations:** ^1^ Department of Public Health, Maseno University, P. O. Box 3275, Maseno 40100, Kenya, maseno.ac.ke; ^2^ Department of Pharmacy, Maseno University, P. O. Box 3275, Maseno 40100, Kenya, maseno.ac.ke

**Keywords:** blood donation, blood donors, body mass index, cardiovascular risk factors, cholesterol, hypertension

## Abstract

**Background:**

Regular blood donation has been associated with lower cardiovascular risk in some studies, possibly through mechanisms involving reduced iron stores and oxidative stress.

**Objective:**

The study investigated the association between blood donation frequency and cardiovascular risk factors among voluntary blood donors.

**Methods:**

The cross‐sectional study examined blood donors classified into regular donors and occasional donors. Blood pressure, body mass index (BMI), fasting blood glucose, and total cholesterol levels were measured. Standard clinical criteria were used to determine the cardiovascular risk factors. Independent *t*‐tests and chi‐square tests were analyzed to compare the two groups. Multivariate logistic regression was applied to control for possible confounding variables.

**Results:**

Regular donors demonstrated lower blood pressure measurements, which included systolic blood pressure of 118 ± 12 mmHg and diastolic blood pressure of 77 ± 8 mmHg. Donors who donated regularly demonstrated a lower BMI of 24.5 ± 3.1 kg/m^2^ compared to donors who donated occasionally. Regular donors showed lower rates of hypertension, 22% compared to 39%, and elevated cholesterol, 21% compared to 35%, with a statistical significance of *p* = 0.04. The study found no differences between the groups regarding their fasting glucose levels or diabetes rates. Regular donors had lower odds of hypertension (OR = 0.52, 95% CI: 0.30–0.89) and elevated cholesterol (OR = 0.56, 95% CI: 0.33–0.95), while the association with overweight/obesity did not reach statistical significance.

**Conclusion:**

Regular blood donation was associated with more favorable cardiovascular risk profiles among donors in this study.

## 1. Introduction

Blood donation serves as an essential component of healthcare systems throughout the world because it supplies essential blood products which hospitals use to treat patients who need transfusions for various medical emergencies [1–3]. Some studies have reported associations between regular blood donation and more favorable cardiovascular profiles [4–7]. The global health crisis of cardiovascular diseases (CVDs) results from multiple risk factors which people can control, including hypertension and obesity and dyslipidemia and diabetes [5, 8, 9].

Previous studies have suggested that frequent blood donation may be associated with lower cardiovascular risk through mechanisms involving reduced iron stores and oxidative stress, both of which have been implicated in atherosclerosis and vascular dysfunction [10, 11]. Different groups of people who donate blood regularly experience better health outcomes compared to those who donate blood less frequently because regular donors show lower blood pressure and better lipid profiles and optimal body mass index (BMI) results [12]. The research results about these connections show multiple inconsistencies. Regular blood donors show better blood pressure and cholesterol and BMI results, but some studies show no changes between them and occasional blood donors [13, 14, 15]. The discrepancies present themselves because researchers used different methods, selected different donors, and created different definitions for donation frequency, while their research results were affected by other variables, including how people lived their lives and what they ate and their economic background.

The available literature on the effects of blood donation frequency only indicates limited information about how blood donation frequency affects cardiovascular risk factors, especially in Kenya and other low‐income countries [11, 16]. Researchers primarily studied donor safety and infection screening methods, while they devoted little research effort to exploring possible cardiovascular effects. The existing knowledge gap prevents organizations from developing effective donor health regulations while they work to improve voluntary donation safety and health advantages. This current study therefore investigated how different blood donation frequencies affected five important cardiovascular risk factors, which included blood pressure, BMI, cholesterol, fasting glucose, and smoking status among voluntary blood donors in Kenya. The study examined whether regular donors who donated two or more times per year showed better cardiovascular health than occasional donors who donated less than two times per year.

## 2. Methods

### 2.1. Study Design

This was a cross‐sectional study to estimate the blood donors’ frequency of donation and cardiovascular risk factors.

### 2.2. Study Population

Eligible criteria included adults aged 18–65 years, at least one blood donation in the previous 12 months, and no prior clinical diagnosis of chronic illnesses such as CVD or diabetes at recruitment. However, participants were screened for undiagnosed diabetes using fasting blood glucose measurements during the study. The exclusion criteria included the following: Donation within the previous 2 weeks, pregnancy or breastfeeding, and contraindications to donation (e.g., active infection and anemia).

### 2.3. Data Collection Procedures

Data collection was performed by using an interviewer‐administered structured questionnaire for the following: Demographics: age, gender, history of illnesses, and current health status; lifestyle factors: smoking and physical activities; blood donation history: frequency and time since last donation. All participants had a standardized physical examination for the following: Blood pressure: Using a calibrated Omron M3 digital sphygmomanometer with the participant seated and after a 5‐min resting period, two readings were taken on the left arm, and the average was recorded. Height and weight: A Seca 213 portable stadiometer and Seca digital scale were used to assess height and weight. Hypertension was defined as systolic BP ≥ 140 mmHg or diastolic BP ≥ 90 mmHg as per JNC 8 guidelines (10). Elevated cholesterol was defined as ≥ 200 mg/dL per NCEP ATP III [5, 17]. Smoking was defined as current daily or occasional use of tobacco products, self‐reported during the interview. Overweight: BMI 25.0–29.9 kg/m^2^; obesity: BMI ≥ 30.0 kg/m^2^. Adjustment was performed for the major confounders; residual confounding by diet or physical activity cannot be ruled out. The classification of donation frequency (≥ 2 vs. < 2 donations per year) was based on commonly used thresholds in donor studies; however, it does not account for long‐term donation patterns or lifetime donation frequency.

### 2.4. Sample Collection and Handling

Venous blood samples (about 5 mL) were collected from each participant using standard aseptic technology into EDTA and plain vacutainer tubes. Serum was separated by centrifugation at 3,000 rpm for 10 min and analyzed on the same day. Total cholesterol was measured using Roche Cobas C111, an automated clinical chemistry analyzer, by the enzymatic colorimetric assay. Fasting blood glucose was measured after an overnight fast of at least 8 h through capillary finger‐prick and measured using an Accu‐Check Active glucose meter with blood. Risk factors were classified as follows: Hypertension: systolic BP ≥ 140 mmHg and/or diastolic BP ≥ 90 mmHg, or the use of antihypertensive drugs. Excess weight/obesity: BMI ≥ 25 kg/m^2^. Elevated cholesterol: Total cholesterol ≥ 200 mg/dL. Diabetes: Fasting glucose ≥ 126 mg/dL or self‐report diagnosis. Smoking: self‐report current tobacco use.

### 2.5. Statistical Analysis

Data were analyzed using IBM SPSS Version 26. Continuous variables were characterized as an average ± standard deviation (SD) and compared using independent *t*‐tests. Classified variables were summarized as frequencies and percentages and compared using a chi‐square test. Multivariate logistic regression was used to assess the association between donation frequency and cardiovascular risk factors. Models evaluating hypertension and elevated cholesterol were adjusted for age, sex, BMI, and smoking status. For the overweight/obesity outcome, BMI was not included as a covariate because it formed part of the outcome definition. The significance was set at *p* < 0.05.

### 2.6. Ethical Considerations

This study strictly adhered to established ethical principles and guidelines for research involving human participants, as outlined in the Declaration of Helsinki.

## 3. Results

### 3.1. Demographic Characteristics of Study Participants

**FIGURE 1:**
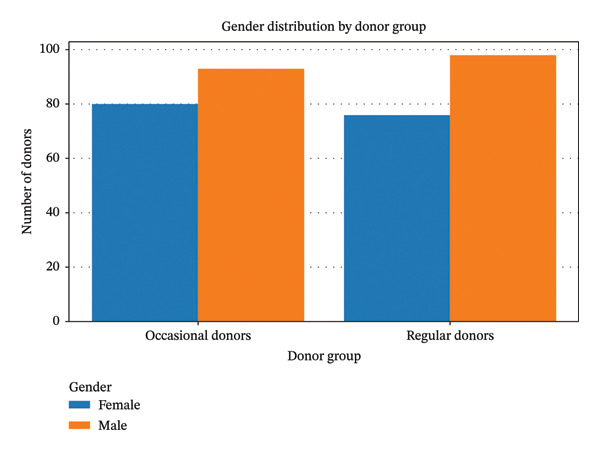
Gender distribution by donor group.

A total of 347 blood donors participated in the study. The average age of the participants was 35.3 years (±5.8), with no significant difference between regular and occasional donors (*p* = 0.31). Gender distribution was balanced, with 55% male and 45% female overall. Regular donors formed 174 participants (50.1%), while the occasional donors included 173 participants (49.9%). Age or gender between these two groups does not have any statistically significant difference, which shows the comparative baseline characteristics (Table 1 and Figure 1).

**TABLE 1:** Demographic characteristics of blood donors.

Characteristic	Total (*n* = 347)	Regular donors (*n* = 174)	Occasional donors (*n* = 173)	*p* value
Age (mean ± SD) (years)	35.3 ± 5.8	34.5 ± 5.4	36.1 ± 6.2	0.31
Male, *n* (%)	191 (55%)	98 (56%)	93 (54%)	0.72
Female, *n* (%)	156 (45%)	76 (44%)	80 (46%)	0.72

### 3.2. Differences in Cardiovascular Risk Factors by Donation Frequency

Blood pressure readings were lower in regular than in those donating irregularly: mean systolic 118 ± 12 mmHg for the former and 126 ± 13 mmHg for the latter, with *p* = 0.03. Diastolic blood pressure was also significantly lower for regular donors, at 77 ± 8 mmHg versus 81 ± 9 mmHg for the occasional donors (*p* = 0.04). There was also a significantly lower BMI for regular donors, 24.5 ± 3.1 kg/m^2^, compared to the BMI of 26.1 ± 4.2 present in occasional donors (*p* = 0.01). Total cholesterol reflected the same trend, with regular donors having a mean of 190 ± 28 mg/dL and occasional donors 202 ± 32 mg/dL (*p* = 0.02) (Table 2).

**TABLE 2:** Cardiovascular risk factors by donation frequency.

Risk factor	Regular donors (*n* = 174)	Occasional donors (*n* = 173)	*p* value
Systolic BP (mmHg)	118 ± 12	126 ± 13	0.03
Diastolic BP (mmHg)	77 ± 8	81 ± 9	0.04
BMI (kg/m^2^)	24.5 ± 3.1	26.1 ± 4.2	0.01
Total cholesterol (mg/dL)	190 ± 28	202 ± 32	0.02
Glucose (mg/dL)	91.2 ± 8.5	92.5 ± 9.1	0.63

*Note:* No statistically significant difference was observed in fasting glucose levels between regular and occasional donors (*p* = 0.63).

### 3.3. Prevalence of Cardiovascular Risk Factors

Hypertension was prevalent among 22% of regular donors, which was significantly lower than the 39% found among occasional donors (*p* = 0.04). Overweight/obesity was less prevalent among regular donors (28%) than among occasional donors (43%), with borderline statistical significance (*p* = 0.05). Elevated cholesterol was found among 21% of regular donors, significantly lower than the 35% found among occasional donors (*p* = 0.03). No significant differences between the groups were observed regarding the prevalence of diabetes (7% vs. 9%, *p* = 0.76) or smokers (14% vs. 16%, *p* = 0.72) (Table 3).

**TABLE 3:** Prevalence of cardiovascular risk factors.

Risk factor	Regular donors (*n* = 174) (%)	Occasional donors (*n* = 173) (%)	*p* value
Hypertension (%)	22	39	0.04
Overweight/obesity (%)	28	43	0.05
Elevated cholesterol (%)	21	35	0.03
Diabetes (%)	7	9	0.76
Smoking (%)	14	16	0.72

*Note:* No statistically significant differences were observed for diabetes or smoking status.

### 3.4. Multivariate Logistic Regression

**FIGURE 2:**
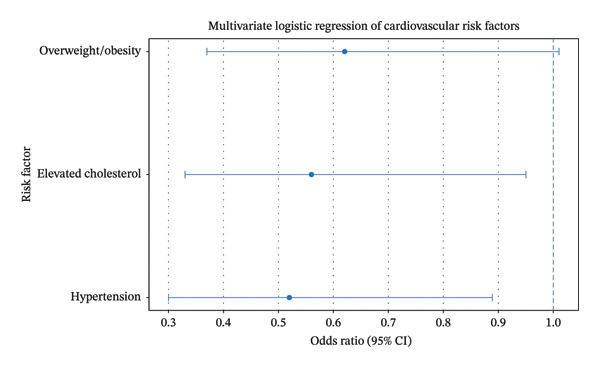
Multivariate logistic regression analysis of cardiovascular risk factors.

After adjusting for age, sex, smoking status, and BMI, regular blood donation was independently associated with lower odds of hypertension (OR = 0.52, 95% CI: 0.30–0.89, *p* = 0.02), overweight/obesity (OR = 0.62, 95% CI: 0.37–1.01, *p* = 0.05), although this association did not reach statistical significance, and elevated cholesterol (OR = 0.56, 95% CI: 0.33–0.95, *p* = 0.03) (Table 4 and Figure 2).

**TABLE 4:** Multivariate logistic regression analysis of cardiovascular risk factors.

Risk factor	Odds ratio (OR)	95% confidence interval (CI)	*p* value
Hypertension	0.52	0.30–0.89	0.02
Overweight/obesity	0.62	0.37–1.01	0.05
Elevated cholesterol	0.56	0.33–0.95	0.03

After adjustment for selected covariates, regular blood donation remained associated with lower odds of hypertension and elevated cholesterol.

## 4. Discussion

The present study assessed the association between blood donation frequency and key cardiovascular risk factors among voluntary blood donors. Regular donors demonstrated lower blood pressure, BMI, and total cholesterol levels compared to occasional donors. The regular donors showed lower rates of hypertension and elevated cholesterol levels, but the connection between their weight and obesity did not show statistical significance after multivariate analysis. The groups showed no significant differences in fasting glucose levels, diabetes rates, or smoking habits. The observed diabetes prevalence likely represents previously undiagnosed cases identified during study screening, rather than known diabetic individuals at enrollment.

The lower blood pressure which regular donors display matches previous research, which found that frequent donors experienced slight decreases in both systolic and diastolic blood pressure [11, 18]. One proposed explanation is that repeated blood donation may influence blood viscosity and vascular function through periodic reduction in blood volume and iron stores. The researchers of the present study worked with cross‐sectional data, which require them to treat their results as links between variables rather than proof of causal relationships.

The regular donors showed lower BMI, which researchers had documented in previous studies that showed frequent donors tend to have better body measurement results [5, 15]. The observation indicates that regular donors tend to develop different health habits because they donate blood more often, which makes them more aware of their health and increases their chances of meeting donation eligibility requirements based on their weight and overall health condition. The phenomenon, which people commonly refer to as the “healthy donor effect,” shows that regular blood donors maintain better health baseline measurements than people in the general population do, according to the authors in [19, 20].

The research found lower total cholesterol levels in regular donors, which matches previous studies that showed blood donation is linked to better lipid profile results according to past findings [21]. One hypothesized mechanism is that repeated phlebotomy may reduce body iron stores, potentially lowering oxidative stress and lipid peroxidation, both of which are implicated in atherosclerosis development. The current study did not measure these mechanisms directly, which leaves them as unproven hypotheses.

The study found no fasting blood glucose differences between regular and occasional donors, nor did it show any difference in diabetes rates between the two groups. Researchers excluded individuals with known diabetes from the study at recruitment, which means the detected diabetes cases in the population represent previously undiagnosed diabetes cases that screening tests found. The study results indicate that blood donation frequency has weak connections to glucose metabolism, which supports previous research showing no connection between donation frequency and glycemic control according to studies [5, 22].

The multivariate analysis demonstrated that regular blood donation was associated with lower odds of hypertension and elevated cholesterol after adjustment for age, sex, smoking status, and BMI. The study found a link between overweight and obesity, but this link did not achieve statistical significance because the confidence interval included the value of one. The observed BMI difference exists because two factors, residual confounding and selection bias, better explain the BMI difference instead of creating an independent relationship.

### 4.1. Limitations

The study design uses cross‐sectional methodology, which limits the establishment of temporal connections between events or proving causal links between variables. The relationship between regular blood donation and cardiovascular risk improvement remains uncertain because it is unknown if healthy individuals donate blood more often than others. The potential influence of the healthy donor effect is likely substantial in this study. Individuals who donate blood regularly are generally healthier, more health‐conscious, and more likely to meet donor eligibility requirements than occasional donors. In addition, important confounding variables including dietary patterns, alcohol consumption, socioeconomic status, and objectively measured physical activity were not comprehensively assessed. Therefore, the observed associations may partly reflect baseline differences in health behaviors rather than an independent effect of blood donation frequency itself. The established cardiovascular risk factors determine cardiovascular risk, which affects the observed results. The donation frequency categories of two or more donations per year and less than two donations per year do not accurately represent long‐term donation patterns, total donation history, or duration since last donation. The study probably led to incorrect classification of exposure, which resulted in reduced capacity to detect actual relationships. Future studies should use comprehensive donation history evidence to establish dose–response pattern connections.

## 5. Conclusion

Regular blood donation was associated with lower blood pressure, BMI, and total cholesterol levels among voluntary blood donors in this study. Regular donors also demonstrated a lower prevalence of hypertension and elevated cholesterol compared to occasional donors. However, because of the cross‐sectional design, healthy donor effect, and potential residual confounding, these findings should be interpreted cautiously and should not be considered evidence of causality. Further longitudinal and mechanistic studies are required to clarify the direction and biological basis of these associations.

## Author Contributions

Collince Odiwuor Ogolla: conceptualization, methodology, investigation, data curation, formal analysis, writing–original draft, writing–review and editing, visualization, project administration, and correspondence.

Bernard Guyah: methodology, supervision, validation, and writing–review and editing.

Apollo O. Maima: conceptualization, supervision, validation, and writing–review and editing.

## Funding

This study did not receive any specific funding from public, commercial, or not‐for‐profit funding agencies.

## Disclosure

All authors have read and approved the final version of the manuscript. The funding source had no role in study design, data collection, analysis, interpretation, manuscript writing, or decision to publish.

## Consent

All authors have given their consent for publication of this article.

## Conflicts of Interest

The authors declare no conflicts of interest.

## Supporting Information

Additional supporting information can be found online in the Supporting Information section.

## Supporting information


**Supporting Information** STROBE checklist (1).

## Data Availability

The authors confirm that the data supporting the findings of this study are available within the article and its Supporting Information. Additional anonymized data may be obtained from the corresponding author upon reasonable request, subject to institutional and ethical approval.
